# Temporal Trends in Tobacco Smoking Prevalence During the Period 2010–2020 in Vietnam: A Repeated Cross-Sectional Study

**DOI:** 10.3389/ijph.2024.1607104

**Published:** 2024-06-27

**Authors:** Lan Thi Hoang Vu, Quyen Thi Tu Bui, Donna Shelley, Raymond Niaura, Bao Quoc Tran, Nga Quynh Pham, Lam Tuan Nguyen, Annie Chu, Angela Pratt, Chi Thi Lan Pham, Minh Van Hoang

**Affiliations:** ^1^ Department of Epidemiology, Hanoi University of Public Health, Hanoi, Vietnam; ^2^ Department of Biostatistics, Hanoi University of Public Health, Hanoi, Vietnam; ^3^Global Center for Implementation Science, School of Global Public Health, New York University, New York, NY, United States; ^4^ Department of Social and Behavioral Sciences, School of Global Public Health, New York University, New York, NY, United States; ^5^ General Department of Preventive Medicine, Ministry of Health, Hanoi, Vietnam; ^6^ World Health Organization, Hanoi, Vietnam; ^7^ Faculty of Sciences, University of British Columbia, Vancouver, BC, Canada; ^8^ Department of Health Policy and Economics, Hanoi University of Public Health, Hanoi, Vietnam

**Keywords:** tobacco smoking, prevalence, Vietnam, trend, socio-economic

## Abstract

**Objectives:** This study used repeated cross-sectional data from three national surveys in Vietnam to determine tobacco smoking prevalence from 2010 to 2020 and disparities among demographic and socioeconomic groups.

**Methods:** Tobacco smoking temporal trends were estimated for individuals aged 15 and over and stratified by demographic and socioeconomic status (SES). Prevalence estimates used survey weights and 95% confidence intervals. Logistic regression models adjusted for survey sample characteristics across time were used to examine trends.

**Results:** Tobacco smoking prevalence dropped from 23.8% in 2010 to 22.5% in 2015 and 20.8% in 2020. The adjusted OR for 2015 compared to 2010 was 0.87, and for 2020 compared to 2010 was 0.69. Smoking decreased less for employed individuals than unemployed individuals in 2020 compared to 2010. Smoking was higher in the lower SES group in all 3 years. Higher-SES households have seen a decade-long drop in tobacco use.

**Conclusion:** This prevalence remained constant in lower SES households. This highlights the need for targeted interventions to address the specific challenges faced by lower-SES smokers and emphasizes the importance of further research to inform effective policies.

## Introduction

Tobacco consumption has significant negative impacts on health, economics, the environment, and society. In 2019, smoking tobacco led to 7.69 million deaths globally and caused a loss of 200 million disability-adjusted life-years [1]. Estimates suggest that smoking contributes to a mortality rate of around 50% in individuals who continue to smoke [2]. Worldwide, there are over 1.3 billion people who use tobacco, with over 84% residing in low- and middle-income countries (LMICs), resulting in a higher burden of tobacco-related illness and death in these regions [3].

As in other developing countries, smoking is a significant public health issue in Vietnam. In 2015, the prevalence of smoking in Vietnam was 22.5% overall, 45.3% among men, and 1.1% among women [4]. It was estimated that deaths attributable to smoking in Vietnam would surpass 50,000 by the year 2023 [5]. In response to this growing public health concern, Vietnam implemented the Law on Prevention and Control of Tobacco Harms in 2012. This law incorporated evidence-based policies aligned with the guidelines of the WHO Framework Convention on Tobacco Control (FCTC) recommendations [6]. These measures included creating smoke-free zones, implementing graphic health warnings on cigarette packs, enforcing a comprehensive ban on tobacco advertising, establishing a smokers’ Quitline in 2015, and introducing the Vietnam tobacco control fund [7]. Some studies in Vietnam showed that these measurements have created changes, such as the decreased prevalence of smoking [4] or the increase of the cigarette excise tax rates from 65% of the ex-factory price to 70% in 2016 and 75% in 2019 [8]. However, limited information is accessible regarding these measurements’ differential effectiveness and enforcement across various sub-populations in Vietnam.

Implementation of FCTC-recommended tobacco policies and interventions has been associated with a significant decline in cigarette consumption and smoking prevalence, primarily in high-income countries [9]. Despite this progress, socioeconomic status (SES) is associated with large disparities in cigarette smoking. Both studies in high developing and LMICs reported that adults with low SES status generally have high cigarette smoking prevalence irrespective of the sociodemographic characteristics of the population [10–13]. These disparities underscore the importance of understanding how tobacco control policies may have impacted tobacco smoking prevalence over time and if changes in tobacco smoking are consistent among different SES groups in Vietnam react to these tobacco control laws over time.

Since 2010, Vietnam has conducted several national representative sample surveys aimed at collecting data on smoking, including two rounds of the Global Adult Tobacco Survey (GATS 2010 and GATS 2015), as well as the STEPs survey in 2020, which assesses non-communicable disease risk factors at the population level. These comprehensive datasets provide a unique opportunity to conduct a repeated cross-sectional study with nationally representative samples. This study aims to use the combined data to analyze the changes in tobacco smoking prevalence over the past decade in Vietnam (i.e., from 2010 to 2020) and to examine the differences in tobacco smoking trends across various demographic and socioeconomic groups.

## Methods

### Data Sources

The Global Adult Tobacco Survey (GATS) is the global standard to systematically monitor adult tobacco use and track key tobacco control indicators. The GATs 2010 data is available at [14]. The GATs 2015 data is available at [15]. The STEPs survey stands for the WHO Stepwise Approach to Surveillance, a framework developed by the World Health Organization (WHO) to collect data on non-communicable disease (NCD) risk factors. The STEPs have three survey stages (STEP 1, 2, and 3), and information about tobacco use was collected in STEP 1. This analysis combined data from 2 GATS surveys in 2010 and 2015 and the STEPs survey in 2020. The detailed information about GATs 2010, 2015, and STEPs 2020 can be found in the previous publications [4, 16, 17].

### Survey Sampling and Questionnaires

All three surveys were conducted using similar methods to generate nationally representative samples with sample sizes calculated to obtain reliable estimates of key variables for sex by urban or rural area. GATS is a nationally representative household survey of adults 15 or older using a standard protocol. A multi-stage, geographically clustered sample design was used to produce nationally representative data. The primary sampling unit was the enumeration area (EA) in the first sampling stage. About 10% of households in each EA were selected in the second stage. One individual was randomly chosen from each participating household to complete the survey. In the GATs 2010, the household response rate was 97.0%, the person response rate was 95.7%, and the overall response rate was 92.8%. There were 9,925 adults aged 15 years and over who completed an interview. In the GATs 2015, 8,996 individual interviews were completed, and the overall response rate was 95.8%. Originally, the target population of the STEPs survey included individuals ages 18–69. However, the STEPs 2020 in Vietnam selected a nationally representative sample of adults aged 15 and above. This survey also employed the same two-stage-random systematic sampling methods as GATs. The sample size for STEP1 included 4,738 subjects (response rate of 94,76%).

The Vietnam GATS in 2010 and 2015 used the standard GATS questionnaire, which collected information on demographic and socio-economic characteristics such as sex, age, ethnicity, education, work status, total household assets, health insurance status, and tobacco smoking. The STEPs 2020 also collected the same demographic and SES information and used the same questionnaire to collect information about tobacco smoking.

### Measures

#### Study Outcomes

Current tobacco smoking: In all three surveys (GATs 2010, GATs 2015, and STEPs 2020), respondents were asked, “Do you currently smoke tobacco on a daily basis, less than daily, or not at all?” Tobacco was defined as cigarette, water pipe, pipe, cigar, or terracotta bowl pipe. Current tobacco smoking includes daily and occasional smokers.

#### Independent Variables

Age group: The age group was categorized as 15–24, 25–44, 45–64, and 65 and over, as these categories are standard in the GAT and STEPS sampling procedures. Geographic location was defined as urban or rural, and marital status as single, married, and other. Socio-economic factors ***:*** Three factors capturing SES were available in the GATs and STEPs surveys. These were the education, employment, and wealth index. Education was a categorical variable, with four categories (i.e., primary education or less, secondary education, and University/college). Employment status had four categories: employed (working for someone else), self-employed, homemaker/students/retired, and unemployed. The Wealth Index is calculated based on household data, including ownership of specific assets like televisions, bicycles, and cars, alongside dwelling attributes such as flooring material, drinking water source type, and toilet and sanitation facilities. It considers characteristics directly associated with wealth status, excluding variables that do not represent assets or related outcomes. All GATs and STEPs surveys used this index as a proxy of household-level wealth [18]. Using the total wealth index score, this study created a wealth quartiles variable by categorizing the total score into quartiles: lowest (<25th), second (25th−50th), third (>50th−75th), and highest (>75th). The validity of the wealth index in measuring the relative wealth of households in Vietnam has been reported elsewhere [19].

### Analysis

Weights were calculated for all datasets to ensure the results represented the entire population. A base weight was first calculated based on the inverse probability of selection, then non-response adjustment was made for non-response at household and individual levels. The SVY procedure in STATA 18 was used to estimate the overall prevalence of tobacco use and their 95% CI for 2010, 2015, and 2020. Survey weights were used for all calculations. The temporal trend in tobacco smoking was examined by analyzing how the prevalence of smoking changes over three time points (the years 2010, 2015, and 2020). The study pooled data from all time points into a single dataset to control the distributions of these characteristics over the different time points. Thus, variables capturing the characteristics of each time point were included in the regression model, allowing for the adjustment of differences in the study population over time. The model also included indicator variables for each time point (year), allowing the analysis of specific changes at each time point. Additionally, the model explored interaction terms between the time indicators and the sample characteristics to ensure that the model could account for any differential effects over time. A *p*-value <0.05 was regarded as statistically significant. As the study examined different measurements of SES, unnecessary adjustment may occur as these variables can be highly correlated [20]. To minimize unnecessary adjustment, each potential covariate was critically assessed for its necessity in controlling confounding by constructing a Directed Acyclic Graph (DAG); variables without theoretical or empirical justification were excluded. Sensitivity analyses were conducted to determine the impact of including or excluding certain covariates on the model’s estimates. For instance, if we hypothesized that education affects the outcome primarily through its effect on occupation, we tested models separately for education and occupation and in combination to ensure that including both did not lead to overadjustment. Thus, the variables controlled in the model were already tested for unnecessary adjustment.

### Ethical Consideration

The paper was based on secondary data from the GATS 2010, 2015, and STEP 2020 with all identifying information removed. All surveys obtained informed consent from the before administering survey questionnaires and testing. All procedures performed in GATs and STEPs involving human participants were in accordance with the ethical standards of The Ethical Review Board for Biomedical Research. The original GATs 2010 and 2015 surveys were approved by the Ethics Committee of the Vietnam Ministry of Health and Hanoi Medical University. The original STEPs 2020 survey was approved by the Hanoi School of Public Health.

## Results

### Characteristics of Study Samples Over Time

Table 1 presents the characteristics of the study samples across three-time points. The percentage of the population living in urban and rural areas did not significantly differ across the time points. The percentage of individuals under 24 years old was lower in 2015 and 2020 compared to 2010 (*p* < 0.001). Additionally, the prevalence of self-employment as an occupational category was highest in the 2020 sample (*p* < 0.001), while the proportion of individuals with a primary school education or less was greatest in the 2010 sample (*p* < 0.001). Regarding marital status, the percentage of single individuals was the lowest in 2020 (*p* < 0.001). For the distribution of the wealth index, the proportion of individuals in the high wealth index (i.e., >75th percentile) was also the lowest in 2020 (*p* < 0.001).

**TABLE 1:** Comparison of study samples over time in Vietnam, period 2010–2020.

Factor	Year 2010 (*N* = 9,925); n(%)	Year 2015 (*N* = 8,996); n(%)	Year 2020 (*N* = 4,738); n(%)
Age group			
15–24	1,656 (16.7)	1,044 (11.6)	365 (7.7)
25–44	4,251 (42.8)	3,434 (38.2)	1,738 (36.7)
45–64	2,886 (29.1)	3,263 (36.3)	2,101 (44.3)
65+	1,132 (11.4)	1,255 (14.0)	534 (11.3)
Sex			
Male	4,356 (43.9)	3,983 (44.3)	2,359 (49.8)
Female	5,569 (56.1)	5,013 (55.7)	2,379 (50.2)
Residence			
Urban	4,958 (50.0)	4,421 (49.1)	2,346 (49.5)
Rural	4,967 (50.0)	4,575 (50.9)	2,392 (50.5)
Marital status			
Single	1,882 (19.0)	1,321 (14.7)	497 (10.5)
Married	7,078 (71.4)	6,483 (72.2)	3,659 (77.5)
Other	959 (9.7)	1,178 (13.1)	564 (11.9)
Occupation			
Employment	1,607 (16.5)	1,672 (19.1)	582 (12.6)
Self-employed	5,637 (57.9)	4,801 (55.0)	2,970 (64.4)
Homemaker/students/retired	2,082 (21.4)	1,748 (20.0)	927 (20.1)
Unemployment	405 (4.2)	516 (5.9)	136 (2.9)
Education			
Primary or less	4,486 (45.2)	3,540 (39.4)	1,841 (38.9)
Lower Secondary	2,672 (26.9)	2,528 (28.1)	1,234 (26.0)
Upper secondary	1,417 (14.3)	1,434 (16.0)	882 (18.6)
College or above	1,346 (13.6)	1,485 (16.5)	781 (16.5)
Wealth Index			
<25th	2,871 (28.9)	2,384 (26.5)	1,463 (30.9)
25–50th	1,735 (17.5)	2,291 (25.5)	1,229 (26.0)
>50–75th	2,303 (23.2)	2,158 (24.0)	1,018 (21.5)
>75th	3,016 (30.4)	2,163 (24.0)	1,018 (21.5)

### The Temporal Trend in Current Tobacco Smoking Prevalence Among all Populations Aged 15 and Over

The prevalence of current tobacco smoking in the respective years 2020, 2015, and 2010 was 19.2%, 22.5%, and 23.8%, respectively. The adjusted odds ratios (ORs) for the year 2015 compared to 2010 were 0.87 (*p* = 0.04), and for the year 2020 compared to 2010, they were 0.69 (*p* < 0.001), indicating a statistically significant decrease in the adjusted prevalence of current tobacco smoking (Table 2).

**TABLE 2:** Prevalence of current tobacco smoking among the population aged 15 and over in Vietnam during the period 2010–2020.

Time	Prevalence	95% CI	OR (CI 95%)	Adjusted OR (CI 95%)
Year 2010	23.78	(22.63; 24.93)	Ref	Ref
Year 2015	22.53	(21.24; 23.81)	0.93 (0.85; 1.03)	0.87 (0.76; 0.99)*
Year 2020	20.78	(19.21; 22.35)	0.84 (0.75; 0.94)**	0.69 (0.60; 0.81)***

Adjusted OR: The adjusted ORs were estimated by applying a multiple logistic regression model, controlling for all the characteristics of the study samples over time, including the distribution of age, sex, residence, marital status, education, employment status, and wealth index.

*p < 0.05; **p < 0.01; ***p < 0.001.

### Temporal Trend of Current Tobacco Smoking by Demographic Characteristics

Rural/Urban Status: Both urban and rural areas exhibited a reduction in the percentage of tobacco smoking over time. However, only a significant decrease in current smoking was observed in rural areas in 2020 compared to 2010 (Table 3).

**TABLE 3:** Changes in smoking prevalence by geographic location, age, and sex in Vietnam, period 2010–2020.

Characteristics	Prevalence of smoking			Comparison between 2015 and 2010	Comparison between 2020 and 2010
	In 2010	In 2015	In 2020	OR (CI 95%)	OR (CI 95%)
By Rural/Urban					
Urban	23.3 (21.9; 24.7)	20.6 (19.0; 22.3)	20.8 (18.2; 23.3)	0.9 (0.8; 0.97)*	0.9 (0.7; 1.03)
Rural	24.0 (22.5; 25.5)	23.5 (21.7; 25.3)	20.8 (18.8; 22.8)	1.0 (0.9; 1.1)	0.8 (0.7; 0.96)*
By age group					
15–24	13.3 (11.2; 15.5)	12.5 (10.0; 15.0)	9.1 (5.7; 12.6)	0.9 (0.7; 1.2)	0.6 (0.4; 1.03)
25–44	28.8 (27.0; 30.55)	26.4 (24.4; 28.4)	21.9 (19.6; 24.3)	0.9 (0.8; 1.01)	0.7 (0.6; 0.8)***
45–64	29.7 (27.5; 31.85)	27.1 (25.1; 29.0)	28.1 (25.6; 30.6)	0.9 (0.8; 1.02)	0.9 (0.8; 1.1)
65+	15.1 (12.6; 17.5)	15.0 (12.7; 17.4)	15.9 (11.7; 20.2)	1.0 (0.8; 1.3)	1.1 (0.7; 1.6)
By sex					
Male	47.4 (45.4; 49.4)	45.3 (43.1; 47.5)	41.1 (38.3; 43.9)	0.9 (0.8; 1.04)	0.8 (0.7; 0.9)***
Female	1.4 (0.9; 2.0)	1.1 (0.7; 1.5)	0.6 (0.3; 0.9)	0.8 (0.4; 1.3)	0.4 (0.2; 0.8)**
By marital status					
Single	15.5 (13.4; 17.6)	17.5 (14.8; 20.2)	12.9 (9.1; 16.8)	1.2 (0.9; 1.5)	0.8 (0.6; 1.2)
Married	28.2 (26.8; 29.7)	25.6 (24.1; 27.1)	23.6 (21.8; 25.4)	0.9 (0.8; 0.97)	0.8 (0.7; 0.9)
Other	10.2 (7.7; 12.7)	10.8 (8.7; 12.9)	13.9 (9.0; 18.8)	1.1 (0.8; 1.5)	1.4 (0.9; 2.3)

*p < 0.05; **p < 0.01; ***p < 0.001.

Age Groups: The youngest group, aged 15–24, exhibited the lowest prevalence of tobacco smoking, yet the change over time only bordered on a significant reduction (p of OR for 2020 compared to 2010 = 0.07). Those 25–44 demonstrated significant declines in tobacco use from 2010 to 2020. No significant changes were observed among those over 44. The prevalence of tobacco smoking was consistently high among age group 45–64, while among age group 65+, this figure was consistently lower across time periods (Table 3).

Sex: Both male and female populations experienced significant decreases in the prevalence of tobacco smoking. Among males, this figure dropped from 47.4% in 2010 to 41.1% in 2020, representing a decrease of 6.3%. Among females, this figure decreased from 1.44% in 2010 to 0.61% in 2020. The absolute change among males was larger than that among females, but the relative changes (as measured by odds ratios) were more substantial among females than males (Table 3).

### Changes in Smoking Prevalence by SES Factors

Education: Analysis of trends based on the highest level of education achieved involved subgroup estimations for four educational attainment categories: primary school or less, lower secondary, upper secondary, and college or above (i.e., Table 4). Generally, a negative association was observed between education level and the prevalence of tobacco smoking, with higher education levels associated with lower smoking prevalence. The trends of tobacco smoking were similar among all groups. All showed a decrease in the prevalence of tobacco smoking in 2020 compared to 2010. However, as shown in Table 4, only the odds ratios (ORs) comparing the years 2020 to 2010 among the population with the highest educational attainment (i.e., college or above) were statistically significant (OR = 0.72, *p* = 0.03).

**TABLE 4:** Changes in smoking prevalence by education, occupation, and wealth index in Vietnam, period 2010–2020.

Characteristics	Prevalence of smoking			Comparison between 2015 and 2010	Comparison between 2020 and 2010
	In 2010	In 2015	In 2020	OR (CI 95%)	OR (CI 95%)
By education					
Primary	26.1 (24.4; 27.8)	26.0 (23.9; 28.1)	24.2 (21.5; 26.9)	1.0 (0.9; 1.1)	0.9 (0.8; 1.1)
Secondary	22.1 (20.2; 24.1)	22.0 (20.0; 24.0)	20.7 (17.8; 23.7)	1.0 (0.9; 1.1)	0.9 (0.8; 1.1)
High school	21.9 (19.2; 24.7)	20.6 (17.8; 23.5)	19.1 (15.5; 22.7)	0.9 (0.7; 1.1)	0.8 (0.6; 1.1)
University	20.3 (17.7; 23.0)	17.3 (14.7; 19.8)	15.5 (12.1; 18.9)	0.8 (0.6; 1.04)	0.7 (0.5; 0.98)*
By occupation					
Employment	23.6 (20.5; 26.6)	19.5 (17.0; 22.0)	13.6 (10.4; 16.7)	0.8 (0.6; 0.99)*	0.5 (0.4; 0.7)***
Free workers	30.9 (29.3; 32.4)	30.4 (28.5; 32.2)	27.6 (25.4; 29.8)	1.0 (0.9; 1.1)	0.9 (0.8; 0.98)*
Home maker/Retired	9.9 (7.9; 11.9)	7.5 (6.0; 9.0)	8.6 (6.0; 11.2)	0.7 (0.5; 1.01)	0.9 (0.6; 1.3)
Unemployment	13.0 (8.8; 17.1)	14.5 (11.0; 18.0)	20.8 (12.6; 29.1)	1.1 (0.7; 1.8)	1.8 (0.95; 3.3)
Students	2.7 (1.4; 3.9)	1.9 (0.5; 3.3)	2.4 (0.03; 4.8)	0.7 (0.3; 1.7)	0.9 (0.3; 2.8)
By wealth index					
<25th	27.0 (24.8; 29.3)	26.9 (24.2; 29.5)	29.1 (26.1; 32.1)	1.0 (0.8; 1.2)	1.1 (0.9; 1.3)
25–50th	25.0 (22.4; 27.6)	23.8 (21.5; 26.2)	24.0 (20.5; 27.5)	0.9 (0.8; 1.1)	0.95 (0.8; 1.2)
>50–75th	23.2 (21.0; 25.5)	20.6 (18.5; 22.7)	18.6 (15.2; 21.9)	0.9 (0.7; 1.03)	0.8 (0.6; 0.98)*
>75th	19.2 (17.5; 21.0)	18.2 (16.0; 20.5)	11.3 (8.9; 13.7)	0.9 (0.8; 1.1)	0.5 (0.4; 0.7)***

*p < 0.05; **p < 0.01; ***p < 0.001.

Employment status: Among the five occupational categories, only two groups demonstrated a statistically significant downward trend over the past decade-specifically, fully employed and self-employed. The prevalence of current tobacco smoking among those in full employment nearly halved in 2020 compared to 2010, dropping from 23.6% to 13.5% (OR = 0.51, *p* < 0.001). For the self-employed, this figure decreased from 30.9% in 2010 to 27.6% in 2020 (Table 4: OR = 0.85, *p* < 0.01). Conversely, the prevalence of current tobacco smoking among the unemployed showed an increasing trend in the last 10 years (i.e., 13% in 2010 to 20.8% in 2020), though at the border of significance (Table 4: OR = 1.77, *p* = 0.07). Among students and homemakers/retirees, the smoking prevalence displayed downward trends, but they were not significant.

Wealth index quartiles: trends were estimated for four quartile groups of wealth index (<25th, 25–50th, >50–75th, and >75th percentiles). Current tobacco smoking prevalence remained higher among those with a lower wealth index. While a significant decreasing trend in tobacco smoking was observed among the high wealth index groups (i.e., 50th percentile or higher), the prevalence of current tobacco smoking among lower wealth index groups (below the 50th percentile) did not show a significant change over time (Table 4). Specifically, the odds ratio (OR) for comparing tobacco smoking between the years 2020 and 2010 was 0.95 (*p* = 0.67) for the group at the 25th–50th percentile and 1.11 (*p* = 0.28) for the groups below the 25th percentile. These ORs were 0.75 (*p* < 0.05) for the wealth index group at the 50th–75th percentile and 0.54 (*p* < 0.001) for the wealth index group above the 75th percentile. Thus, not only was the prevalence of current tobacco smoking lower among higher wealth index groups, but the decreasing trends were also more pronounced in these higher wealth index groups. In the multiple regression model, adjusting for the differences in the characteristics of the study samples over time—such as age, sex, residence, marital status, and education, the interaction between time and wealth index quartiles was statistically significant across wealth index groups. This significant interaction underscores that, even after adjusting for differences in sample characteristics over time, the trends in current tobacco smoking varied among the four wealth index groups.

## Discussion

This study analyzed repeated cross-sectional data from three National surveys in Vietnam to assess the changes in tobacco smoking prevalence and associated trends across diverse subgroups.

Overall, the prevalence of tobacco smoking among adults in Vietnam has decreased by 3% over the last decade (from 23.78% to 20.78%). Comparing these findings to global trends, the World Health Organization (WHO) reports a decrease in current tobacco use prevalence in the Southeast Asia Region from around 50% in 2000 to 29% in 2020 [21]. It’s worth noting that despite the relatively small decrease, Vietnam’s overall tobacco use prevalence is lower than that of many other countries. With a lower baseline prevalence, achieving further declines becomes more challenging. Thus, while the change in Vietnam may seem modest, it carries significant importance, particularly in the context of implementing the Tobacco Control Law in Vietnam since 2013. Furthermore, this study reveals a noteworthy trend: while there was a non-significant decrease in tobacco smoking prevalence from 2010 to 2015, a significant and consistent decline emerged between 2010 and 2020. These changes, though not uniform across all subgroups of the Vietnamese population, indicate a positive trajectory in tobacco control efforts.

Age groups exhibited distinctive trends in smoking prevalence, with individuals under 44 years old showing more substantial declines in smoking over the studied period. This finding was similar to data reported in other countries [22] and may be explained by the fact that prevention efforts often focus on educating and discouraging tobacco use among adolescents and young adults, potentially contributing to a larger decline in smoking prevalence in these age groups. Younger generations, exposed to more comprehensive anti-smoking campaigns and educational initiatives, may be likely to adopt healthier behaviors and more likely to quit smoking [23]. It was also possible that younger individuals face more barriers to obtaining tobacco products due to stricter enforcement of regulations, higher prices, or reduced exposure to tobacco advertising [24].

The significant sex disparity in tobacco smoking in Vietnam emphasized the need to implement policies and programs that are sensitive to sex differences. By tailoring interventions to meet the needs of men, enhancing enforcement in male-dominated areas, and conducting further research [25], Vietnam can make more significant strides toward reducing the overall smoking rates and mitigating the health impacts associated with tobacco use.

SES disparity in smoking behaviors in literature refers to the association between lower SES and higher prevalence of smoking [11, 12, 26]. In previous studies, SES could be measured by education, income, or occupation. Overall, the association between lower education and lower income with higher use of tobacco products was reported across different countries, regions, races/ethnicities [11–13]. This study examines the SES disparity in smoking by examining the variations over time for all three factors capturing SES level. In our study, all educational categories displayed a reduction in smoking prevalence by 2020 compared to 2010, yet higher education correlated with lower smoking prevalence. Previous studies attributed the link between higher education and lower smoking prevalence to several factors, including increased awareness of health risks, better access to resources, healthier social networks, and improved decision-making skills [27–29]. Regarding occupation, full employment, and self-employment categories demonstrated significant declines in smoking prevalence, in contrast to a concerning increase among the unemployed population over the decade (from 13% in 2010 to 17.1% in 2020), although this increase was not statistically significant. This upward trend in smoking among the unemployed in Vietnam can be attributed to various factors, including shifts in health behaviors linked to their unemployment status, limited access to health education programs [30–32], and the relatively lower cost of tobacco in Vietnam [33].

The study also examined variations in trends of current smoking across four quartile groups of the household wealth index. Wealth index disparities were evident, with lower wealth index groups consistently exhibiting higher smoking prevalence. While higher wealth index groups showed decreasing trends, prevalence remained relatively stable among lower wealth index groups, exacerbating existing socioeconomic disparities in tobacco smoking. Prior research has consistently highlighted a link between lower socioeconomic status (SES) and heightened tobacco product use [11, 12, 26], coupled with a decreased likelihood of quitting [34]. These elements contribute to the widening gaps between high and low SES over time. The resulting socioeconomic disparity in smoking may contribute to increased economic and health burdens for those with lower SES, potentially adding to existing health and socioeconomic inequalities [12, 26].

This study adds to the literature by demonstrating a widening of SES-related disparities over time in an LMIC that has implemented recommended tobacco-related policies and programs. The integration of data from three national surveys [35, 36] facilitated the creation of a repeated cross-sectional database and allowed for the generation of robust and nationally representative estimates of smoking behaviors over time, as well as the examination of smoking trends among different sub-population groups. However, limitations arose due to the lack of specific details provided in the original survey data regarding the factors that can explain SES disparities in smoking, such as access to resources to quit smoking, social network, mental health status … Thus, while this study provides insights into smoking trends, it was not capable of identifying the complete array of factors influencing these trends. Future studies employing longitudinal designs and appropriate comparisons (e.g., across areas with/without implementation of legally mandated tobacco control measures) could offer a more comprehensive understanding of the causal relationship between specific tobacco-related policies and programs on smoking behavior and factors influencing SES-related disparities in tobacco use.

The Tobacco Control Law in Vietnam, effective from 1 May 2013, serves to govern various measures aimed at reducing demand, limiting supply, and implementing preventive actions against tobacco-related harm. This legislation extensively regulates tobacco goods’ production, importation, distribution, and sale-purchase. Notably, it mandates health warnings on tobacco packaging, promotes tobacco cessation initiatives, and enforces increased taxes on tobacco products [7]. The study revealed a significant, gradual decline in the prevalence of current smokers in Vietnam over a decade, potentially influenced by the implementation of FCTC-recommended tobacco policies and interventions. However, growing disparities across socioeconomic groups highlight the need for targeted policies and smoking cessation interventions to address these gaps in smoking behaviors.

## Data Availability

The datasets used and/or analyzed during the current study are available from the corresponding author upon reasonable request.
